# An in vitro study of the effect of 5-ALA-mediated photodynamic therapy on oral squamous cell carcinoma

**DOI:** 10.1186/s12903-020-01239-8

**Published:** 2020-09-16

**Authors:** Ying Ma, Shujuan Qu, Liangpeng Xu, Hongbo Lu, Baoguo Li

**Affiliations:** Department of Stomatology, Linyi Central Hospital, No.17 Jiankang Road, Yishui County, Linyi, 276400 Shandong China

**Keywords:** 5-ALA, Photodynamic, Oral squamous cell carcinoma, In vitro, Action, Mechanism

## Abstract

**Background:**

The primary aim of this study was to observe the effect of 5-ALA-mediated photodynamic therapy on oral squamous cell carcinoma in vitro.

**Methods:**

SCC25 cells were divided into the observation group and the blank control group. Different concentrations of 5-ALA and SCC25 cells were co-incubated for different times, and the concentration of protoporphyrin IX was detected by flow cytometry. SCC25 cells were divided into the 5-ALA group (100 mg/L), the laser irradiation group alone, the 5-ALA plus laser irradiation group, and the blank control group (0 mg/L 5-ALA), and the methyl thiazolyl tetrazolium (MTT) solution method was used (each group was incubated for 4, 8 and 12 h in turn). The cell survival rate was calculated. Using annexin V-fluorescein isothiocyanate/propidium iodide method, the apoptosis of SCC25 cells was detected by flow cytometry.

**Results:**

The level of protoporphyrin IX in SCC25 cells increased with increased concentrations of 5-ALA and length of incubation. However, after 12 h, protoporphyrin IX level in SCC25 cells was gradually stabilized, and similar effect was obtained with 100 mg/L or more 5-ALA, indicating that the level of protoporphyrin IX in SCC25 cells was determined by 5-ALA concentration and incubation time. 5-ALA plus laser irradiation exerted an inhibitory effect on the growth of SCC25 cells, which was highly associated with drug dose and incubation time. Compared with the control group, laser irradiation alone or 5-ALA alone had no effect on the apoptosis of SCC25 cells. Different concentrations of 5-ALA combined with laser irradiation showed a remarkable effect of apoptosis, and a higher apoptosis rate was seen with higher drug concentrations.

**Conclusion:**

5-ALA-mediated photodynamic therapy affects the growth of SCC25 cells in vitro, which may provide a new idea for the clinical treatment of oral squamous cell carcinoma.

## Background

Oral squamous cell carcinoma (OSCC) is the one of the most common malignant tumors with a high prevalence rate and a poor prognosis, which poses a serious threat to the life and health of patients. At present, surgery, adjuvant radiotherapy and chemotherapy are regarded as the main approaches to treat OSCC [1, 2]. As the conventional surgical method has some limitations, it cannot be widely used. On the other hand, radiotherapy and chemotherapy have toxic and side effects to some extent, and cancer cells could be induced to produce drug resistance during the operation, which reduces the efficacy of chemotherapy. Therefore, there has been a high demand for clinical research in recent years to adopt safe, effective and less limited treatment methods for OSCC. Photodynamic therapy is a therapeutic approach using photosensitive drugs combined with light irradiation to selectively destroy cancer cells under the effect of photodynamic process [3]. The main advantages of this therapy include its non-invasive or minimally invasive nature, selective effect on cancer cells, lack of toxic side effects and lack of drug resistance, and this approach can be used in the treatment of OSCC. Photosensitizer, laser irradiation and oxygen play an important role in the photodynamic process. The possible mechanism is that the photosensitizer leads to the generation of energy through specific laser irradiation and the mobilization of surrounding oxygen molecules, resulting in the formation of reactive oxygen species. Reactive oxygen species leads to oxidative damage of cancer cells and induce apoptosis or necrosis of cancer cells [4]. 5-aminolevulinic acid (5-ALA) is the second-generation photosensitizer and an endogenous 5-carbon compound, which can produce strong photosensitive protoporphyrin IX. It can have photodynamic effect after laser irradiation. 5-ALA-mediated photodynamic therapy can specifically kill cells or cancer cells that proliferate too fast in the tissue, without affecting normal tissues. Given that protoporphyrin IX has quick metabolism, there is no need to avoid light for a long time after treatment [5]. The current study was conducted to observe the effect of 5-ALA-mediated photodynamic therapy on OSCC in vitro, with an attempt to explore its underlying role and mechanism.

## Methods

### Materials and instruments

5-ALA, fetal bovine serum (FBS), 2′,7′-dichlorodihydrofluorescein diacetate, and MTT (Sigma) were used. Our study also utilized the following materials and instruments: enzyme labeling instrument produced by Bio-Rad Biomedical Products (Shanghai) Co., Ltd., Dulbecco’s modified Eagle medium (DMEM) by Shanghai Yuanpei Biotechnology Co., Ltd., semiconductor laser by Beijing Bingshou Technology Co., Ltd., annexin V-fluorescein isothiocyanate/propidium iodide apoptosis detection kit by BD Co., flow cytometer by BD Co.; ribonuclease A by Beyotime Biotechnology Co., Ltd., human OSCC cell line SCC25 from ATCC that was cultured at 37 °C, 5% CO_2_.

### Photodynamic therapy parameters

The treatment setting was: 87 mW/cm^2^ power, 635 nm irradiation wavelength, 10.4 J/cm^2^ energy density.

### Detection of protoporphyrin IX in SCC25 cells

SCC25 cells were divided into the observation group and the blank control group. Different concentrations of 5-ALA (5, 10, 25, 50, 100 and 150 mg/L) and the observation group cells were co-incubated for different lengths of time (2, 4, 6, 12 and 24 h).

The growth of protoporphyrin IX in SCC25 cells was observed by flow cytometry after treatment with 5-ALA. SCC25 cells in logarithmic growth phase were evenly spread in a 12-well plate at 8 × 10^4^ cells per well, and 1 mL DMEM containing 15% FBS was added to each well and cultured for 24 h. After removal of the culture medium containing serum, the supernatant was removed; after wash with sterile phosphate buffer for two times, DMEM (without fetal bovine serum) was used to prepare 5-ALA. The concentrations were set as 5 mg/L (the 5 mg/L group), 10 mg/L (the 10 mg/L group), 2 5 mg/L (the 25 mg/L group), 50 mg/L (the 50 mg/L group), 100 mg/L (the 100 mg/L group), 150 mg/L (the 150 mg/L group) and 0 mg/L (the blank control group). 5-ALA and SCC25 cells were incubated for 2, 4, 8, 12 and 24 h, and the concentration of protoporphyrin IX in cells was detected by flow cytometry. The intracellular average fluorescence intensity was analyzed by C Flow Plus 1.0.264.15 software by BD Co. (USA).

### MTT was used to detect the cell activity

SCC25 cells were divided into the 5-ALA alone group (100 mg/L), the laser irradiation alone group, the 5-ALA combined with laser irradiation group (5-ALA concentration was set from low to high as 5, 10, 25, 50 and 100 mg/L), and the blank control group (0 mg/L 5-ALA).

Activity of SCC25 cells was detected by MTT after treatment with 5-ALA combined with laser irradiation. SCC25 cells in the logarithmic growth phase were evenly spread in a 96-well plate at 6 × 10^3^ cells per well, and 100 μL of DMEM containing 15% FBS was added to each well and cultured for 24 h. The supernatant was removed. After wash with sterile phosphate buffer for two times, the Dulbecco’s modified Eagle medium (without fetal bovine serum) was used to prepare 5-ALA. After co-incubation of 5-ALA and SCC25 for 4, 8 and 12 h, the cells were irradiated by laser. After the supernatant was discarded, 100 μL DMEM containing 15% FBS was added to each well. After 24 h of culture, 20 μL MTT solution was added to each well to stop the culture. The cells were incubated in the dark for 4 h, and then the supernatant was removed. Then, 150 μL dimethyl sulfoxide was added, and mixed for 8 min. Absorbance was read at 490 nm, and the cell survival rate was calculated as follows:

$$ {\displaystyle \begin{array}{c}\mathrm{Cell}\ \mathrm{survival}\ \mathrm{rate}=\mathrm{the}\ \mathrm{absorbance}\ \mathrm{value}\ \mathrm{of}\ \mathrm{the}\ \mathrm{study}\ \mathrm{group}/\mathrm{the}\ \mathrm{absorbance}\ \mathrm{value}\ \mathrm{of}\ \mathrm{the}\ \mathrm{blank}\\ {}\mathrm{control}\ \mathrm{group}\times \kern0.37em 100\%.\end{array}} $$

### Apoptosis was detected by flow cytometry

SCC25 cells were divided into the 5-ALA alone group (100 mg/L), the laser irradiation alone group, the 5-ALA combined with laser irradiation group (5-ALA concentration was set from low to high as 5, 10, 25, 50 and 100 mg/L), and the blank control group (0 mg/L 5-ALA).

After co-incubation of 5-ALA and SCC25 cells for 12 h, the cells were irradiated by laser, and then cultured in DMEM with 15% FBS for 24 h. Apoptosis of SCC25 cells was induced by 5-ALA combined with laser irradiation, and apoptosis was detected by annexin V-fluorescein isothiocyanate/propidium iodide double staining. Five μL annexin V-fluorescein isothiocyanate/propidium iodide were added. After 5 min, the apoptosis of SCC25 cells was detected by flow cytometry. The experiment was done in triplicate.

### The effect of 5-ALA on SCC25 cell cycle was detected by flow cytometry

Cells in the logarithmic growth phase were placed in a 6-well plate at a density of 5 × 10^5^ mL^− 1^ and cultured at 37 °C for 24 h. The cells were divided in two groups: the 5-ALA (100 mg/L) combined with laser (2 h) group and the blank control group. After 24 h, the cells were collected and washed twice with sterile phosphate buffer twice. The single cell suspension was fixed with 70% ethanol, the temperature was set at 4 °C, and the fixed solution was washed with sterile phosphate buffer. Then, 100 μ L ribonuclease A was added at 37 °C water bath for 30 min, and mixed with 400 μL propidium iodide solution in the dark at 4 °C. After 30 min, the results were detected and recorded by flow cytometry. The above experiments were repeated 3 times in each group to ensure the accuracy of the data.

### Statistical analysis

Data were expressed as $\overline{x}\pm s$ and analyzed by IBM Microsoft SPSS 21.0 software. Repeated measurement analysis of variance was used for overall comparison, t-test was used for pairwise comparison of inter-group and intra-group data. Categorical data was expressed as rate (%), and chi-square χ^2^ test was used for comparison. A *P* < 0.05 represented significant statistical difference.

## Results

### Comparison of average fluorescence intensity of intracellular protoporphyrin IX in SCC25 cells of different groups with different concentrations of 5-ALA for different lenths of time

Table 1 shows that the level of protoporphyrin IX in SCC25 cells increased with increases in 5-ALA concentration and prolongation of time. However, after 12 h, the protoporphyrin IX level in SCC25 cells were gradually stabilized, and similar effect was obtained after using 100 mg/L or more 5-ALA, indicating that the level of protoporphyrin IX in SCC25 cells was determined by 5-ALA concentration and time.

**Table 1:** 5-ALA triggered OSCC cells to produce protoporphyrin IX ($\overline{x}\pm s$, × 10^3^)

Group (5-ALA)	2 h	4 h	6 h	12 h	24 h
Blank	0.007 ± 0.001	0.005 ± 0.001	0.008 ± 0.002	0.018 ± 0.003	0.026 ± 0.003
5 mg/L	0.238 ± 0.006^*^	0.361 ± 0.012^*^	0.351 ± 0.017^*^	0.693 ± 0.037^*#^	2.673 ± 0.048^#^
10 mg/L	0.612 ± 0.061^*^	0.816 ± 0.028^*^	0.735 ± 0.123^*#^	1.487 ± 0.067^*#^	5.432 ± 0.079^*#^
25 mg/L	2.557 ± 0.161^*^	3.212 ± 0.037^*^	5.721 ± 0.483^*#^	11.987 ± 0.654^*#^	16.987 ± 2.391^*#^
50 mg/L	4.962 ± 0.127^*^	7.791 ± 0.263^*^	11.723 ± 1.273^*#^	21.076 ± 0.763^*#^	27.473 ± 3.473^*#^
100 mg/L	7.643 ± 1.673^*^	11.376 ± 2.065^*#^	14.723 ± 1.238^*#^	26.753 ± 2.632^*#^	32.763 ± 3.973^*#^
150 mg/L	8.124 ± 1.721^*^	12.451 ± 2.376^*#^	16.302 ± 1.098^*#^	29.876 ± 2.398^*#^	33.902 ± 4.872^*#^

The sample size at each time point was 3 in each group; compared with the blank group at the same time point, *P < 0.05; compared with the same group after 2 h, #*P* < 0.05

### Comparison of the survival rate of SCC25 cells treated with 5-ALA and laser for different time between groups

Laser irradiation alone, or 5, 10, 25, 50 or100 mg/L 5-ALA exerted no effect on cell viability, and the cell survival rate was 100%. 5-ALA combined with laser irradiation inhibited the growth of SCC25 cells, which was highly associated with drug concentration and time. Laser irradiation after co-incubation of 5-ALA with SCC25 cells for 12 h showed significant cytotoxic effect. When the concentration of 5-ALA was 50 mg/L, the cell survival rate was (8.97 ± 2.97) mg/L (Table 2).

**Table 2:** Comparison of the survival rate of SCC25 cells treated with 5-ALA and laser for different time between group ($\overline{x}\pm s$,%)

Group (5-ALA)	2 h	4 h	6 h	12 h
Blank	102.01 ± 7.03	98.16 ± 6.93	97.37 ± 6.51	95.97 ± 6.19
5 mg/L	98.08 ± 7.17	94.09 ± 6.09	87.92 ± 7.89	81.92 ± 4.99
10 mg/L	95.83 ± 6.83	93.81 ± 6.19	89.36 ± 4.87	75.82 ± 3.91^*#^
25 mg/L	82.13 ± 3.01^*^	76.89 ± 3.98^*^	66.87 ± 8.79^*#^	37.92 ± 8.73^*#^
50 mg/L	62.01 ± 8.02^*^	57.93 ± 8.35^*^	41.28 ± 7.32^*#^	8.97 ± 2.97^*#^
100 mg/L	78.82 ± 6.53^*^	53.73 ± 4.83^*^	29.76 ± 5.83^*#^	7.19 ± 1.26^*#^

The sample size at each time point was 6 in each group; compared with the blank group at the same time point, *P < 0.05; compared with the same group after 4 h, #*P* < 0.05

### Effects of 5-ALA combined with laser irradiation on apoptosis and necrosis of SCC25 cells in each group

Compared with the blank control group, laser irradiation alone and 5-ALA alone showed no effect on the apoptosis of SCC25 cells, and no significant difference was found in the early apoptosis rate, late apoptosis rate and necrosis rate of SCC25 cells (*P* > 0.05). Different concentrations of 5-ALA combined with laser irradiation showed a significant effect on apoptosis. A higher apoptosis rate was found with higher drug concentrations, which was significantly higher than that in the control group (*P* < 0.05) (Table 3).

**Table 3:** Effect of 5-ALA combined with laser irradiation on apoptosis rate and necrosis rate of SCC25 cells in each group ($\overline{x}\pm s$, %)

Group (5-ALA)	Early apoptosis rate	Late apoptosis rate	Necrosis rate
Blank	0.49 ± 0.07	0.33 ± 0.11	0.39 ± 0.19
100 mg/L	0.51 ± 0.13	0.44 ± 0.17	0.55 ± 0.26
Laser	0.63 ± 0.17	0.35 ± 0.08	0.48 ± 0.23
Laser + 5 mg/L	1.41 ± 0.36^*^	3.09 ± 0.31^#^	1.38 ± 0.34^*^
Laser + 10 mg/L	1.18 ± 0.35^*^	5.29 ± 0.49^#^	2.73 ± 0.28^#^
Laser + 25 mg/L	7.83 ± 0.98^#^	17.87 ± 3.01^#^	7.41 ± 1.23^#^
Laser + 50 mg/L	5.47 ± 0.76^#^	24.54 ± 4.02^#^	13.89 ± 2.01^#^
Laser + 100 mg/L	24.87 ± 4.01^#^	44.28 ± 6.73^#^	14.83 ± 3.03^#^

The sample size at each time point was 3 in each group; compared with the blank group at the same time point, *P < 0.05; compared with the same group after 4 h, #*P* < 0.05

### Effect of 5-ALA combined with laser irradiation on SCC25 cells

Significant difference was observed in the effect of 5-ALA combined with laser irradiation compared with the blank control group (*P* < 0.05) (Table 4).

**Table 4:** Effect of 5-ALA combined with laser irradiation on SCC25 cell cycle ($\overline{x}\pm s$)

Group/Stage (%)	I	II	III	IV
Blank	55.832 ± 0.213	3.2876 ± 0.098	5.987 ± 0.211	39.765 ± 0.238
100 mg/L 5-ALA+Laser	78.932 ± 0.238^#^	2.473 ± 0.029^#^	4.087 ± 0.231^#^	17.231 ± 0.029^#^

The sample size of each group was 6, compared with group E, #*P* < 0.05

## Discussion

OSCC is one of the most common oral malignant tumors. With approximately 140,000 people dying of oral cancer each year, this condition poses a serious threat to human health. OSCC is located in the face; hence, routine surgery will affect normal pronunciation, chewing, swallowing and other oral functions. Meanwhile, facial loss can lead to negative psychological impact for patients who receive the treatment [6, 7]. Photodynamic therapy has superior advantages in the treatment of OSCC due to its non-invasive or minimally invasive characteristics [8]. Photodynamic laser tissue penetration has some limitations, which is only available for superficial parts of the body, such as facial lip tumor, tongue tumor and so on, so 5-ALA-mediated photodynamic therapy may be the best choice for the treatment of OSCC [9, 10]. Now 5-ALA-mediated photodynamic therapy has been used in the treatment of actinic keratosis and superficial basal cell carcinoma with beneficial effects. However, through literature review, there are limited studies concerning the use of 5-ALA-mediated photodynamic therapy in the treatment of OSCC, and its specific role and mechanism remain largely unclear. Therefore, the present study aimed to explore the effect and underlying mechanism of 5-ALA-mediated photodynamic therapy on OSCC in vitro.

Photodynamic therapy is a new approach for targeted treatment of cancer, which is ideally suited for solid cancer, benign tumors and some precancerous lesions [11–13]. In clinical treatment, the recommended dose of photosensitizer has no cytotoxicity, but photoactivation with the participation of oxygen can induce cytotoxicity. The second-generation photosensitizer has been used in the clinic, which is not only specific to cancer tissue, but also has a killing effect in the laser irradiated area. Additionally, photosensitizer in normal tissue can disappear within 12 to 24 h, so it causes no harm to normal tissue, which is regarded as the greatest advantage of photodynamic therapy for cancer. 5-ALA is the second-generation photosensitizer in photodynamic therapy. It is synthesized by the action of δ-amino- ƴ -ketovalerate synthetase on succinyl-CoA and glycine, which is the precursor of heme synthesis in vivo [14]. 5-ALA has no photosensitivity, but it can produce strong photosensitizer protoporphyrin IX after a series of transformations in cells. Related clinical studies have confirmed that 5-ALA can greatly increase the content of protoporphyrin IX in tissues to the peak within 3 h, which slowly decreases after 6 h and disappears after 24 h. However, with different sensitivities of 5-ALA to photodynamic process, the amount of 5-ALA absorbed by different types of cancer cells is different; hence, there are different degrees of protoporphyrin IX in the cells [15]. According Wang et al. [16], the fluorescence intensity of 5-ALA is not the same in OSCC cells with different degrees of differentiation. The results of this study showed that the level of protoporphyrin IX in SCC25 cells was determined by 5-ALA concentration and time, which is consistent with previous studies. Our results also found that the best 5-ALA incubation time was not the same as other studies, and the reason may be that cytology had no correlation. Based on aggregated results, 5-ALA-mediated photodynamic therapy is available for the treatment of OSCC. Additionally, compared with the first-generation photosensitizer, protoporphyrin IX has a shorter half-life in the body, so the photodynamic mediated by 5-ALA shows better biosafety, which is able to remarkably shorten treatment time, thus reducing light exposure for patients.

Based on our results, 5-ALA combined with laser irradiation could significantly inhibit the proliferation of SCC25 cells in vitro. When the mass concentration of 5-ALA was 50 mg/L, the cell survival rate was (8.97 ± 2.97) mg/L. Different concentrations of 5-ALA combined with laser irradiation showed remarkable effect on apoptosis, and higher drug concentrations caused more apoptosis. After the intervention of 5-ALA combined with laser irradiation, the proportion of grade I SCC25 cells was significantly higher than that of the blank control group, the proportion of grade IV SCC25 cells was lower than that of the blank control group, and that of grade II and III was slightly lower than that of the the blank control group. A novel study reported that in 5-ALA-mediated photodynamic therapy, Bcl-2, p-Akt, p-mTOR and iNOS were up regulated in OSCC cells, suggesting an activation of anti-apoptosis and cell proliferation pathways [17]. The above results confirmed that 5-ALA-mediated photodynamic therapy offers the potential to regulate the growth of OSCC in vivo and in vitro, and is linked to the regulation of metabolism and proliferation of SCC25 cells. It also indicates that 5-ALA-mediated photodynamic therapy has dual effects and is not controllable, and its clinical implementation needs to be further verified.

In addition, as a minimally invasive or non-invasive therapy, 5-ALA-mediated photodynamic therapy can specifically select cancer cells and effectively shorten treatment time to reduce light exposure. Nevertheless, limitation exists considering that the therapy fails to go deep into the cancer tissue; hence, it is insufficient to kill cancer tissue, and it can cause damage to the normal tissue. Therefore, the latest research is performed to combine fractionated light with iron chelating agent, with an attempt to provide a new insight for OSCC treatment.

## Conclusion

In conclusion, 5-ALA-mediated photodynamic therapy inhibits the growth of SCC25 cells in vitro, which may provide a new idea for OSCC treatment.

## Data Availability

The datasets generated and analyzed during the current study are available from the corresponding author on reasonable request.

## Abbreviations

OSCC: Oral squamous cell carcinoma
5-ALA: 5-aminolevulinic acid
